# Mg‐MOF‐74 Derived Defective Framework for Hydrogen Storage at Above‐Ambient Temperature Assisted by Pt Catalyst

**DOI:** 10.1002/advs.202401868

**Published:** 2024-03-09

**Authors:** Shiyuan Liu, Yue Zhang, Fangzhou Zhu, Jieyuan Liu, Xin Wan, Ruonan Liu, Xiaofang Liu, Jia‐Xiang Shang, Ronghai Yu, Qiang Feng, Zili Wang, Jianglan Shui

**Affiliations:** ^1^ Tianmushan Laboratory Hangzhou 310023 China; ^2^ School of Materials Science and Engineering Beihang University Beijing 100191 China; ^3^ Department of Applied Biology and Chemical Technology The Hong Kong Polytechnic University Hong Kong Hong Kong SAR 999077 China; ^4^ School of Reliability and Systems Engineering Beihang University Beijing 100191 China

**Keywords:** chemisorption, defective MOF, hydrogen storage, Mg‐MOF‐74, techno‐economic analysis

## Abstract

Metal–organic frameworks (MOFs) are promising candidates for room‐temperature hydrogen storage materials after modification, thanks to their ability to chemisorb hydrogen. However, the hydrogen adsorption strength of these modified MOFs remains insufficient to meet the capacity and safety requirements of hydrogen storage systems. To address this challenge, a highly defective framework material known as de‐MgMOF is prepared by gently annealing Mg‐MOF‐74. This material retains some of the crystal properties of the original Mg‐MOF‐74 and exhibits exceptional hydrogen storage capacity at above‐ambient temperatures. The MgO_5_ knots around linker vacancies in de‐MgMOF can adsorb a significant amount of dissociated and nondissociated hydrogen, with adsorption enthalpies ranging from −22.7 to −43.6 kJ mol^−1^, indicating a strong chemisorption interaction. By leveraging a spillover catalyst of Pt, the material achieves a reversible hydrogen storage capacity of 2.55 wt.% at 160 °C and 81 bar. Additionally, this material offers rapid hydrogen uptake/release, stable cycling, and convenient storage capabilities. A comprehensive techno‐economic analysis demonstrates that this material outperforms many other hydrogen storage materials at the system level for on‐board applications.

## Graphical abstract

A novel, highly defective Mg‐MOF‐74 is reported, featuring linker vacancies that enhance hydrogen chemisorption strength at neighboring MgO_5_ nodes. Consequently, defective metal‐organic framework (MOF) demonstrates the capacity to absorb significant amounts of dissociated and undissociated hydrogen at temperatures up to 160 °C. This approach is anticipated to expedite the utilization of MOFs in hydrogen storage applications.

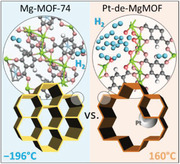

## 1 Introduction

Hydrogen, as a clean energy carrier, necessitates advanced storage techniques for safe and efficient utilization, particularly in transportation applications. In comparison to gaseous and liquid hydrogen storage techniques, solid‐state hydrogen storage presents a compelling alternative due to its superior safety feature and reduced spatial requirement.^[^ ^1^ ^]^ Many hydrides, such as LaNi_5_, MgH_2_, and LiBH_4_, have shown great potential for hydrogen storage through a chemisorption mechanism.^[^ ^2^ ^]^ Porous materials, on the contrary, utilize physisorption for molecular hydrogen storage and require high pressure and extremely low temperatures to optimize storage capacity.^[^ ^3^ ^]^ However, the ongoing challenge lies in achieving both high capacity and moderate operating temperatures simultaneously.^[^ ^4^ ^]^

Metal‐organic frameworks (MOFs) are promising candidates for hydrogen storage, with hydrogen storage capacities of 2‒9 wt.% at 77 K and 10 MPa.^[^ ^5^ ^]^ The high refrigeration costs necessitate an elevation in operating temperatures. The known strategies involve desolvation,^[^ ^6^ ^]^ ligands or cation modification,^[^ ^7^ ^]^ and additional metal species incorporation,^[^ ^8^ ^]^ which enhance the adsorption strength of MOFs to hydrogen. The absorption enthalpies have been thereby increased to the level of chemical adsorption which is sometimes called nondissociated chemisorption.^[^ ^4^, ^9^ ^]^ Another approach utilizes spillover catalysts to facilitate the dissociation/recombination of hydrogen, thereby enabling the storage of hydrogen atoms in MOFs.^[^ ^10^ ^]^ The above strategies allow MOFs to store considerable amounts of hydrogen (0.7‒2.5 wt.%) at ambient temperatures, which is a big advancement in MOF hydrogen storage.^[^ ^11^ ^]^ However, it is worth noting that previous reports have also shown that when the operating temperature rises and exceeds the ambient temperature, the hydrogen storage capacity of these MOFs will decrease, which poses a safety issue.^[^ ^12^ ^]^ This problem particularly occurs in transportation scenarios. For example, a MOF hydrogen storage tank adsorbs a large amount of hydrogen at ambient temperature. When exposed to summer sunlight for a long time, the stored hydrogen will be released due to the increased ambient temperature, which leads to a significant and undesired increase in the internal pressure of the MOF tank. Therefore, it is necessary to further enhance the hydrogen adsorption strength of MOFs to improve the safety of hydrogen storage systems, which may also increase the hydrogen storage capacity of MOFs.^[^ ^13^ ^]^

Here, we report a highly defective framework, abbreviated as de‐MgMOF, as an above‐ambient temperature hydrogen storage material. The synthesis process is very simple just by mild annealing of Mg‐MOF‐74 in Ar/H_2_ (95%/5%), which creates a substantial number of linker vacancies while retaining some crystal properties of the original MOF. The resultant coordinatively unsaturated MgO_5_ knots near these vacancies present strong hydrogen adsorption strength, elevating the hydrogen adsorption temperature of de‐MgMOF to much above ambient levels. With the help of trace amounts of Pt catalyst, the materials of Pt‐de‐MgMOF achieve a high reversible capacity of 2.55 wt.% at an appropriate temperature of 160 °C and a pressure of 80 bar. This material also exhibits fast hydrogen uptake/release kinetics and excellent cycling stability, as well as tolerance to natural storage environments. System‐level techno‐economic analysis further underscores the advantages of Pt‐de‐MgMOF over conventional room‐temperature and low‐temperature hydrogen storage materials for onboard applications.

## 2 Results and Discussion

### 2.1 Preparation and Structure of De‐MgMOF

**Figure 1:**
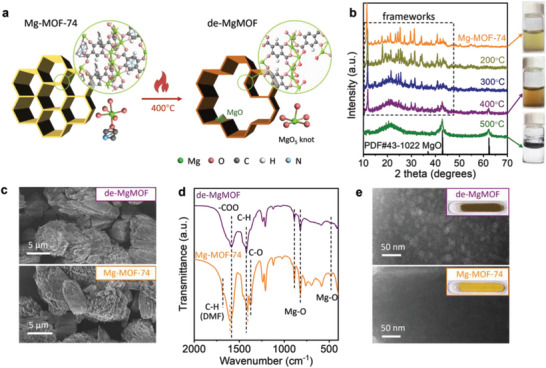
a) Schematic illustration of the construction of de‐MgMOF by mild annealing of Mg‐MOF‐74 in Ar/H_2_ (95%/5%). b) XRD patterns of Mg‐MOF‐74 and thermal‐treated samples. The digital photographs of the dissolution results of samples are displayed on the right. c) SEM images, d) FT‐IR spectra, and e) TEM and photo images of de‐MgMOF and Mg‐MOF‐74.

The preparation of de‐MgMOF is schematically illustrated in **Figure** **1**a. Mg‐MOF‐74 has hexagonal channels, in which Mg atoms are positioned at vertex positions in a zigzag manner. Each Mg atom coordinates with five O atoms (MgO_5_ sites) and dangles a DMF or H_2_O guest molecule.^[^ ^14^ ^]^ Based on the results of X‐ray diffraction (XRD), thermogravimetric analysis (TGA), differential scanning calorimetry (DSC), and digital photographs (Figure 1b; Figures S1 and S2, Supporting Information), it can be seen that the Mg‐MOF‐74 would not completely carbonize until 500 °C in Ar/H_2_ (95%/5%). According to the hydrogen storage capacity of the annealed samples (Figure S3, Supporting Information), the optimal heating temperature was determined to be 400 °C. At 400 °C, the guest molecules were removed according to Fourier transform infrared spectroscopy (FT‐IR, Figure S4, Supporting Information) result, and nuclear magnetic resonance (NMR, Figure S5, Supporting Information) also proved the partial linker degradation. The annealed sample inherits the particle morphology (Figure 1c; Figure S6, Supporting Information) and high surface area of original Mg‐MOF‐74 (1015 m^2^ g^−1^ of 400 °C‐treated sample versus 1262 m^2^ g^−1^ of Mg‐MOF‐74, Figure S7, Supporting Information). Although the crystal structure is partially destroyed, the main feature is preserved according to the remaining diffraction peaks in Figure 1b. This can be further proved by a dissolution experiment. The 400 °C‐treated sample was still soluble in water like the pristine Mg‐MOF‐74; while the carbonized sample (treated at 500 °C) was insoluble. The distinct reduction in BET surface area, especially the surface area of the micropore, indicates the complete destruction of the MOF structure (Figure S8, Supporting Information). In addition, the Mg‐O signals at 485, 824, and 890 cm^−1^ in the FT‐IR spectrum also demonstrate the preservation of the MgO_5_ knots in the 400 °C treated sample (Figure 1d). Notably, the Mg─O signal of the treated sample did not shift compared to the pristine MOF, because the removed ligands were replaced by oxygen after exposure to air as demonstrated later.

The reduction and shift of the CO and COO peaks at 1374 and 1603 cm^−1^ evident in the defective structure in de‐MgMOF.^[^ ^15^ ^]^ This is also supported by the electron paramagnetic resonance (EPR) result as shown in Figure S9 (Supporting Information). At last, transmission electron microscopy (TEM) reveals the formation of abundant mesopores in the 400 °C‐treated sample due to the loss of some linkers (Figure 1d; Figure S10, Supporting Information). Unfortunately, the linker vacancy cannot be directly observed using our microscopy as MOF‐74 is highly sensitive to the electron beam.^[^ ^16^ ^]^ The decomposed linkers were transferred to small MgO nanoparticles, as detected by XRD. These MgO nanoparticles cannot be observed under TEM due to their small size and low contrast. Based on the above‐mentioned altered framework structure and preserved crystalline features, the 400 °C‐treated Mg‐MOF‐74 can be regarded as a defective MOF, denoted as de‐MgMOF in the following discussion.

### 2.2 Hydrogen Storage Properties at Above‐Ambient Temperature

**Figure 2:**
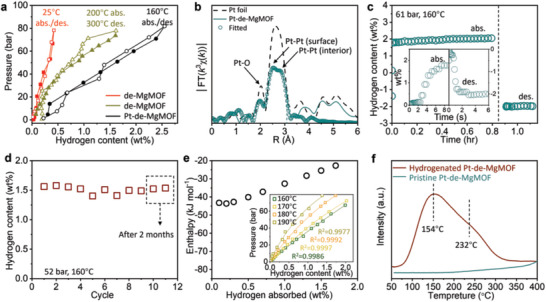
a) PCT curves of de‐MgMOF and Pt‐de‐MgMOF at indicated adsorption/desorption temperatures. b) *k^3^*‐weighted Fourier transform of EXAFS spectra at Pt L‐edge of Pt‐de‐MgMOF. c) Hydrogen adsorption and desorption curves at 160 °C and 61 bar, and d) cycling test at 160 °C and 52 bar of Pt‐de‐MgMOF. The last two cycles were performed after the sample was stored in a glass vial for 2 months in air, at 26 °C and 60% relative humidity. To shorten the test time, the cycling test was carried out under a reduced pressure of 52 bar so that the absorption could reach equilibrium within 20 min. e) Calculated enthalpy change as a function of the amount of the adsorbed hydrogen. Inset: Experimental H_2_ content (dots) and isotherm fits (lines) of Pt‐de‐MgMOF calculated using an exponential model. f) H_2_‐TPD curves of hydrogenated and pristine Pt‐de‐MgMOF.

From the pressure‐composition‐temperature (PCT) curve in **Figure** **2**a, it can be seen that de‐MgMOF has a reversible hydrogen storage capacity of 0.4 wt.% at 25 °C and 70 bar, higher than the 0.1 wt.% of the pristine Mg‐MOF‐74 (Figure S11, Supporting Information). Interestingly, the H_2_ uptake of de‐MgMOF jumps to 1.6 wt.% when the adsorption and desorption temperatures increase to 200 and 300 °C, respectively. This phenomenon indicates that de‐MgMOF has typical chemisorption characteristics, similar to the hydrogen absorption/desorption processes of nano‐sized Mg.^[^ ^17^ ^]^

To reduce the operation temperature, we applied a hydrogen spillover catalyst of Pt on de‐MgMOF (denoted as Pt‐de‐MgMOF). Pt is in the forms of single atoms and clusters as observed by high‐angular annular dark‐field scanning transmission electron microscopy (HAADF‐STEM, Figure S12, Supporting Information), with content of only 0.35 wt.% in Pt‐de‐MgMOF as measured by inductively coupled plasma optical emission spectrometer (ICP‐OES, Table S1, Supporting Information). The EDS mapping shows that Pt is uniformly distributed over de‐MgMOF (Figure S13, Supporting Information). The *k^3^*‐weighted Fourier transforms of EXAFS at Pt L‐edge (Figure 2b) show a split Pt─Pt peak, suggesting the presence of Pt─Pt bonds with different lengths. According to literature reports, there exist short bonds on the surface and long bonds on the inside of platinum clusters.^[^ ^18^ ^]^ In addition, the size of Pt clusters is estimated to be 2–3 nm based on the coordination number simulation (Table S2, Supporting Information),^[^ ^19^ ^]^ which is consistent with the STEM observation. According to the hydrogen absorption curve at 25 °C (Figure S14, Supporting Information) and previous studies,^[^ ^20^ ^]^ the spillover effect should not occur at room temperature. When the absorption/desorption temperatures increase to 160 °C, the hydrogen uptake of Pt‐de‐MgMOF jumps to 2.55 wt.% at 81 bar, achieving a breakthrough in the hydrogen storage capability of MOFs at above ambient temperature (Figure 2a; Table S3, Supporting Information). Two other batches of Pt‐de‐MgMOF were synthesized to reproduce the high hydrogen storage performance (Figure S15, Supporting Information). Notably, the PCT curves of Pt‐de‐MgMOF exhibit a proportional relationship between hydrogen storage capacity and pressure. This proportionality arises from the material's noncrystalline structure, which enables nonstoichiometric hydride formation.

The kinetics of Pt‐de‐MgMOF is very fast at 160 °C. About 80% of hydrogen can be adsorbed within 8 s and 90% of hydrogen can be desorbed within 4 s (Figure 2c). This can be attributed to the fully exposed adsorption sites in the framework without hydrogen diffusion resistance. Also due to this property, Pt‐de‐MgMOF possesses stable cycling ability as shown in Figure 2d. XRD patterns, N_2_ sorption‐desorption isotherms, and SEM and TEM images of the samples before and after cycling indicate that Pt‐de‐MgMOF has excellent structural stability during cycling (Figures S16–S19, Supporting Information). Furthermore, the performance of Pt‐de‐MgMOF would not degrade even after 2 months of storage in a vile ambient environment, showing excellent storage stability of the material.

As shown in Figure 2e, the adsorption enthalpy of Pt‐de‐MgMOF ranges from −22.7 to −43.6 kJ mol^−1^, calculated from the fitted PCT curves using the Clausius−Clapeyron equation. See Figure S20 and Table S4 (Supporting Information) for the fitting plots and parameters. These enthalpy values are much higher than that of physisorption (typically ≈−5 kJ mol^−1^) and a little lower than that of Mg nanoparticles (e.g., −59.5 kJ mol^−1^).^[^ ^21^ ^]^ The adsorption entropy of Pt‐de‐MgMOF ranges from −80.8 to −118.9 J K^−1^ mol^−1^ (Figure S21, Supporting Information), much lower than −135 J K^−1^ mol^−1^ of MgH_2_.^[^ ^22^ ^]^ The relatively low entropy means that the hydrogen in de‐MgMOF retains a certain degree of freedom,^[^ ^23^ ^]^ which contributes to its fast kinetics. The temperature programmed desorption (TPD) measurements show that the hydrogen desorption of Pt‐de‐MgMOF starts from 50 °C and reaches the peak at 154 °C. A part of the hydrogen is released at 232 °C (Figure 2f). About 0.6 wt.% of residual hydrogen can be figured out from the sample exposed to air based on the integration of TPD peaks (Table S5, Supporting Information). The activation energy (*E_a_*) of Pt‐de‐MgMOF hydrogenation was calculated to be 70.6 kJ mol^−1^ (Figure S22, Supporting Information), close to that of Mg nanoparticles (80 kJ mol^−1^) and much larger than that of previously reported chemisorption MOFs (16–37 kJ mol^−1^).^[^ ^9^, ^24^ ^]^ All these thermodynamic and kinetic data indicate that there exist several types of hydrogen adsorption strengths in Pt‐de‐MgMOF, and some of them are quite strong.

### 2.3 Hydrogen Storage Mechanism

**Figure 3:**
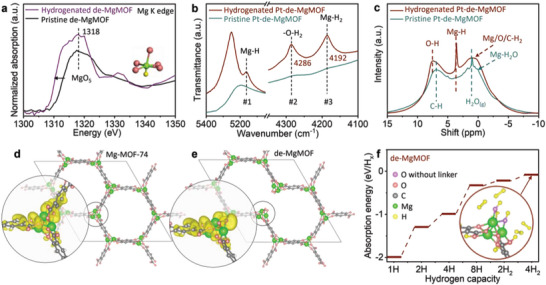
a) XANES spectra at Mg K edge of pristine and hydrogenated de‐MgMOF. b) NIR and c) ^1^H NMR spectra of hydrogenated and pristine Pt‐de‐MgMOF. d,e) Structure and charge accumulation diagram of the MgO_5_ knots in Mg‐MOF‐74 and de‐MgMOF. The charge accumulation region is colored in yellow with an isovalue of 0.2 × ^−3^e Å^‐1^. f) Calculated hydrogen adsorption energies of de‐MgMOF. Inset: the corresponding configuration of 8 hydrogen atoms and 4 hydrogen molecules adsorbed on a defective region.

X‐ray absorption near edge structure (XANES) spectra of pristine and hydrogenated de‐MgMOF are carried out in **Figure** **3**a without the interference of Pt. Mg K edge absorption shows a negative shift on the hydrogenated sample, indicating the reduction of Mg valance due to the adsorption of hydrogen on MgO_5_.^[^ ^25^ ^]^ The Mg 1s XPS spectra and FT‐IR (Figures S23 and S24, Supporting Information) also demonstrate that the hydrogenated Pt‐de‐MgMOF contains Mg‐H, consistent with the previous studies of the heterolytic H_2_ dissociation on MgO.^[^ ^17^, ^26^ ^]^ The near‐infrared (NIR, Figure 3b) spectrum shows a distinct Mg─H absorption as indicated by peak #1. The appearance of peak #2 indicates the presence of molecular H_2_ near MgO_5_ sites through ─O─H_2_ bonds. The appearance of peak #3 indicates the presence of molecular H_2_ on Mg atoms (─Mg─H_2_).^[^ ^6b^ ^]^^1^H NMR spectra also detect atomic H (the chemical shift at 3.6 ppm, Mg─H; the chemical shift at 7.5 ppm, O─H) and molecular H_2_ (the chemical shift at −0.5 to 3.1 ppm, Mg/O/C─H_2_) in hydrogenated Pt‐de‐MgMOF (Figure 3c).

Density functional theory simulations are performed to deepen the understanding of the hydrogenation of de‐MgMOF. The configurations and the positively charged regions of the desolvated Mg‐MOF‐74 and the de‐MgMOF are shown in Figures 3d,e, respectively. The configurations of hydrogen adsorption on desolvated Mg‐MOF‐74 are shown in Figure S25 (Supporting Information), with the hydrogen adsorption energy (*E*_ads_) of −0.159 eV/H_2_. This absorption strength is stronger than physisorption, similar to the result of a previous simulation study.^[^ ^27^ ^]^ Compared to Mg‐MOF‐74, the Mg on de‐MgMOF loses more electrons, while the O gains more electrons (Figure S26, Supporting Information), which increases the binding strength to H atoms for both sites.^[^ ^28^ ^]^ Moreover, the charged O can polarize H_2_ molecules, which helps the material to adsorb H_2_ molecules to increase the hydrogen storage capacity.^[^ ^29^ ^]^ Further calculations are made to determine the configurations of hydrogen adsorption on a defective Mg_3_O_9_ cluster. The first introduced H_2_ molecule spontaneously dissociates into two H atoms that are adsorbed onto O atoms. The activation of an H_2_ molecule on the Mg_3_O_9_ cluster is illustrated in Figure S27 (Supporting Information) with a high energy barrier of −1.984 eV/H. Up to 8 H atoms can combine with a Mg_3_O_9_ cluster, where 2 H atoms are bound to Mg and 6 H atoms are bound to O, with adsorption energy of −1.30 and −0.33 eV/H, respectively. This behavior is similar to the heterolytic H_2_ dissociation on MgO as reported in the literature.^[^ ^30^ ^]^ The unique property of the Mg_3_O_9_ cluster is attributed to the additional adsorption of H_2_ molecules on the more negatively charged O. The overall *E*_ads_ of de‐MgMOF lies between −2.00 eV/H and −0.09 eV/H_2_, indicating a mild chemisorption strength (Figure 3f). Figure S28 (Supporting Information) shows how the Pt catalyst facilitates the H_2_ dissociation and the H diffusion on the material.

It is worth mentioning that the desolvated MgO_5_ sites would readily adsorb O_2_ or H_2_O from the environment upon exposure to air.^[^ ^31^ ^]^ This can be verified by the increased valence of Mg as detected by XANES in Figure S29 (Supporting Information). *E*_abs_ is as high as −0.009 eV/H_2_ for a MgO_5_ site carrying an H_2_O guest molecule (Figure S30, Supporting Information), which explains why the as‐synthesized Mg‐MOF‐74 was only able to adsorb hydrogen at ultra‐low temperature (such as −196 °C) in previous reports.^[^ ^32^ ^]^ Fortunately, the adsorbed O_2_ or H_2_O molecules can be removed by activation before use. This feature enables de‐MgMOF to be stored in an ambient environment, which is meaningful to the practical application of this material.

### 2.4 Techno‐Economic Analysis

**Figure 4:**
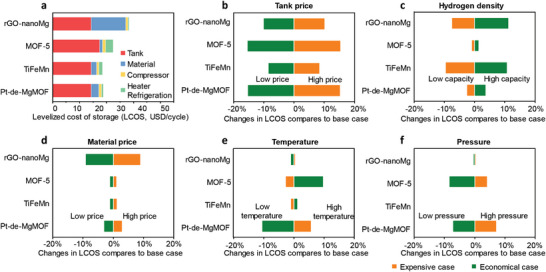
a) Cost breakdowns of hydrogen storage materials. b–f) Sensitivity analysis and comparison of Pt‐de‐MgMOF to other hydrogen storage materials in the system level. Both the upper and lower bounds of the analysis are subjected to a 20% vibration with respect to the base case value.

To assess the economic potential of Pt‐de‐MgMOF for use in light‐duty fuel cell vehicles, we performed the techno‐economic analysis and compared with three representative hydrogen storage materials, including a traditional room‐temperature hydrogen storage material TiFeMn,^[^ ^33^ ^]^ Mg nanocrystals encapsulated by reduced graphene oxide (rGO‐nanoMg), and a physisorption hydrogen storage material MOF‐5.^[^ ^34^ ^]^ Due to space limitations, several other promising materials, such as Ti‐based alloys, have not been included in this study. Considering the safety and kinetics requirements for fuel cell vehicles, hydrides like bulk MgH_2_ and LiBH_4_ with high operation temperatures (above 400 °C) are not considered.^[^ ^35^ ^]^ **Figure** **4**a displays the results of the levelized cost of storage (LCOS). LCOS represents the total cost of the hydrogen storage system, including the tank, the storage material, and the energy consumption for the hydrogen compressor and heater/refrigeration. For the calculation details see Supporting Information. The LCOS of TiFeMn is 18.6 USD/cycle, which is promising for industrial application, due to the moderate hydrogen absorption requirements, relatively high hydrogen storage capability, and cost‐effective nature. Nano Mg has been the subject of extensive research in recent years.^[^ ^17^, ^36^ ^]^ rGO‐nanoMg has a high hydrogen uptake of 6.5 wt.% and outstanding operational characteristics. However, its LCOS is still as high as 28.4 USD/cycle due to the expensive synthesis of nano‐Mg. MOF‐5 has a high hydrogen density and low material cost, but its operation temperature is too low, which leads to a high LCOS of 22.6 USD/cycle. In comparison, Pt‐de‐MgMOF demonstrates a competitive LCOS of 18.8 USD/cycle,^[^ ^37^ ^]^ due to its low Pt dosage and subsequently reduced costs, alongside its moderate operation temperature and pressure.

The sensitivity analysis was also carried out to evaluate the extent to which each characteristic has an impact on LCOS.^[^ ^38^ ^]^ The factors that closely relate with the onboard application of hydrogen storage systems are chosen for this study (Figure S31, Supporting Information). The critical factors include tank cost, gravimetric hydrogen density at the material level, raw material cost for the storage material, and operation temperature and pressure. For solid hydrogen storage systems, the cost of the tank still significantly affects the total economics of hydrogen storage systems. It is estimated that as technology advances and production quantities rise, tank prices will decrease.^[^ ^39^ ^]^ Therefore, the LCOSs of MOF‐5 and Pt‐de‐MgMOF, which are sensitive to tank price, will be lowered more than metal hydrides (Figure 4b). LCOSs of MOF‐5 and Pt‐de‐MgMOF are insensitive to the hydrogen storage density (Figure 4c). This is undoubtedly favorable given the unavoidable performance decline. It means that even if the material‐level hydrogen storage density decreases, the system cost will not increase significantly. The LCOS sensitivity of Pt‐de‐MgMOF is also quite low to the material price (Figure 4d). The solvent recovery and liquid‐assisted grinding could further reduce the costs and environmental hazards.^[^ ^40^ ^]^ As shown in Figures 4e,f, the LCOS of Pt‐de‐MgMOF is sensitive to the operation temperature and pressure. It is therefore expected to be further reduced the cost by means of efficient catalysts. In contrast, these working conditions are difficult to optimize for MOF‐5.^[^ ^41^ ^]^

## 3 Conclusion

In summary, we've developed a highly defective framework material capable of chemisorption of hydrogen at above‐ambient temperatures. Key hydrogen adsorption sites are coordinatively unsaturated MgO_5_ knots near linker vacancies. These knots have much higher hydrogen adsorption strength than their desolvated counterparts, leading to high working temperatures of 200–300 °C. With the assistance of a spillover catalyst (Pt) to reduce the operation temperature to an appropriate level, the material of Pt‐de‐MgMOF exhibits a remarkable hydrogen storage capability of 2.6 wt.% at 160 °C. Moreover, this material has excellent cyclability and can be maintained in ambient. A system‐level techno‐economic analysis highlights the competitiveness of Pt‐de‐MgMOF. Our study pioneers the use of MOF‐derived materials for high‐capacity hydrogen storage beyond ambient temperatures.

## Conflict of Interest

The authors declare no conflict of interest.

## Supporting information

Supporting Information

## Data Availability

The data that support the findings of this study are available from the corresponding author upon reasonable request.
