# Supplementary material for: Mg‐MOF‐74 Derived Defective Framework for Hydrogen Storage at Above‐Ambient Temperature Assisted by Pt Catalyst
Source: Adv Sci (Weinh). 2024 Mar 9;11(18):2401868. doi: 10.1002/advs.202401868 (PMC11095220; doi:10.1002/advs.202401868)
Supplement: Supplementary file 1 — Supporting Information [file ADVS-11-2401868-s001.pdf]

ADVANCED SCIENCE
Open Access

Supporting Information

for *Adv. Sci.*, DOI 10.1002/advs.202401868

Mg-MOF-74 Derived Defective Framework for Hydrogen Storage at Above-Ambient Temperature Assisted by Pt Catalyst

*Shiyuan Liu, Yue Zhang, Fangzhou Zhu, Jieyuan Liu, Xin Wan, Ruonan Liu, Xiaofang Liu, Jia-Xiang Shang, Ronghai Yu, Qiang Feng, Zili Wang and Jianglan Shui**

Supporting Information

Mg-MOF-74 Derived Defective Framework for Hydrogen Storage at Above-Ambient Temperature Assisted by Pt Catalyst

Shiyuan Liu, Yue Zhang, Fangzhou Zhu, Jieyuan Liu, Xin Wan, Ruonan Liu, Xiaofang Liu, Jia-Xiang Shang, Ronghai Yu, Qiang Feng, Zili Wang, and Jianglan Shui*

AUTHOR INFORMATION

Shiyuan Liu, Jianglan Shui

Tianmushan Laboratory, Hangzhou 310023, China

E-mail: shuijianglan@buaa.edu.cn

Shiyuan Liu, Fangzhou Zhu, Jieyuan Liu, Xin Wan, Ruonan Liu, Xiaofang Liu, Jia-Xiang Shang, Ronghai Yu, Jianglan Shui

School of Materials Science and Engineering, Beihang University, Beijing 100191, China

Shiyuan Liu

Department of Applied Biology and Chemical Technology, The Hong Kong Polytechnic University, Hong Kong SAR 999077, China

Yue Zhang, Qiang Feng, Zili Wang

School of Reliability and Systems Engineering, Beihang University, Beijing 100191, China

Experimental section

Material preparation

Synthesis of Mg-MOF-74. The original Mg-MOF-74 is synthesized using the previously reported methods. Briefly, 0.2 g (1.0 mmol) of 2,5-dihydroxyterephthalic acid (DHTP) was dissolved in 5.0 g of N, N-dimethylformamide (DMF) to form a yellow solution. Then the solution is added dropwise to a $Mg(NO_3)_2$ solution (2.6 mmol in 5.0 g DMF). The mixture is placed in an oven at 120°C for 20 h, after which the solid is recovered by filtration. The solid is washed twice with 20 mL of methanol and dried in a vacuum oven at 60°C for 48 h to obtain Mg-MOF-74.

Synthesis of Pt-Mg-MOF-74. The sample preparation for the addition of hydrogen spillover catalyst Pt is performed by adding 0.5 wt% (calculated by mass of Pt) K_2PtCl_4 to $Mg(NO_3)_2$ solution. The remaining steps are the same as the synthesis of pristine Mg-MOF-74, to obtain sample Pt-Mg-MOF-74. The exact catalyst addition is detected by ICP-OES.

Synthesis of Pt-de-MgMOF. The above dried Mg-MOF-74 are placed in crucibles and heated at 400°C for 4 h in a tube furnace, under the Ar/H_2 (95%/5%) atmosphere. The heating rate and cooling rate are 10 °C min^{-1}. After cooling to room temperature, the ocher-yellow powders are collected and denoted as de-MgMOF. The heating temperature for Pt-Mg-MOF-74 is 400°C and the resulting sample is denoted as Pt-de-MgMOF.

Characterization

The X-ray diffraction (XRD) data of the samples are obtained using Rigaku D/max 2500, and the morphology is analyzed using scanning electron microscope SEM (JSM7500, JEOL), TEM (JEM-2100F, JEOL) and HAADF-STEM (FEI Titan Cubed Themis G2 300). Thermogravimetric analysis (TGA) and simultaneous differential scanning calorimetry (DSC) were performed using the analyzer STA-449F3 under the atmosphere of Ar. Samples were loaded and weighed in air and the ramp rates were set to 10 °C min^{-1}. EXAFS results were acquired at beam line 1W1B of the Beijing Synchrotron Radiation Facility. The samples were prepared as paraffin pellets, and the data fitting were further conducted by using the Athena program, Artemis program, and IFEFFIT codes. Electron paramagnetic resonance (EPR) experiments were performed on a Bruker A300-10/12 spectrometer in 297–433 K temperature range. All the EPR experiments have been performed on powder samples. Hydrogen desorption was detected using temperature-programmed desorption (TPD) spectroscopy (Autohchem II 2920), with a heating rate of 5 K min^{-1} and a carrier gas of N_2 (flow rate of 30 cm^3 STP min^{-1}). The composition and elemental valence were analyzed using X-ray photoemission spectroscopy (XPS, Thermo Escalab 250Xi). The material structure and the hydrogen adsorption configurations FT-IR were examined by an infrared spectrometer (Thermo Fisher, Nicolet 6700) and NIR were examined by a near-infrared spectrometer (Thermo Fisher, Nicolet Antaris II). The precise quantity of Pt and Mg was examined by inductively coupled plasma optical emission spectroscopy (ICP-OES, Optima-7000DV). The binding mode of hydrogen was detected by nuclear magnetic resonance spectrometer (NMR, Bruker AVANCE III 600 M). The magnetic field strength is 14.09 T, and the chamber cavity diameter is 54 mm. The surface area of each sample was measured by N_2

sorption isotherms (SSA-7000, Builder) at 77 K using the Brunauer–Emmett–Teller (BET) method, and the porosity parameters were analyzed using the software QuadraWin (version 6.0).

Performance test

Hydrogen storage measurement. Hydrogen adsorption and desorption kinetics, cycling stability, and isothermal adsorption/desorption curves were performed using the automatic sieverts-type high-pressure adsorption instrument (GASpro, Setaram, accuracy 1%R) and a manual sieverts-type pressure–composition–temperature (PCT) setup. For GASpro the tests are carried out in a sample cell volume of approximately 15.1 mL by using high-purity hydrogen gas (99.999%). The real gas state equation is applied for the calculation. The samples were loaded and weighed in air with the sample mass of 150-200 mg. Pre-treatment before test consisted of heating at 100°C for 30 min in vacuum. The sample holder volume is ~15 mL, the volume calibration was conducted using He gas for five times, and the average number is the final result. Samples were activated once by introducing 3 MPa H_2 gas and holding at 250°C for 30 min, followed by evacuating for 30 min. The determination for equilibrium in PCT test was the fluctuation of less than 0.002% in pressure value within 1 min. For cyclic test, a dynamic vacuum of 30 min was arranged before each cycle of hydrogen absorption to ensure complete dehydrogenation of the sample. The standard Pd powder was used for calibration to prevent the systematic errors and influence of the temperature gradient between the sample cell (298-598 K) and gas reservoir (303 K).

Enthalpy and entropy calculations. The PCT curves are fitted by using exponential fits, the differential enthalpies of adsorption (ΔH) and entropies of adsorption (ΔS) were calculated using the Clausius–Clapeyron relationship in equation (1), where R is the ideal gas constant, P is the pressure, and T is the temperature.

$$lnP = -\frac{\Delta H}{R}\left(\frac{1}{T}\right) + \frac{\Delta S}{R} \qquad (1)$$

Activation energy of desorption. The Kissinger equation is applied for the calculation of the activation energy (E_a). The validity of this method has been thoroughly described previously[1] and is represented as equation (2), where β is the heating rate, A is the frequency factor, R is the ideal gas constant, and T_P absolute peak temperature.

$$ln\left(\frac{\beta}{T_P^2}\right) = ln\frac{AR}{E_a} - \frac{E_a}{R} \times \frac{1}{T_P} \qquad (2)$$

DFT calculation.

Pristine structure of Mg-MOF-74 with the additional solvent molecule was obtained in the Cambridge Structural Database. Upon activation, the solvent molecule will be removed, leaving a primitive rhombohedral unit cell. The defective structure was obtained by removing a linker from the unit cell. The hydrogenated structure was obtained by adding H_2 molecule or H atom randomly into the channel. All framework atoms are relaxed during the structural optimization in this work. As for the hydrogen absorption calculation, only adsorbing hydrogen are relaxed while the framework atoms are fixed because of the minor effect of the framework flexibility. The adsorption amount on one defective MgO_5 site is assumed to be the same as that on the others.

All geometric optimization and energy calculations were performed in the Vienna ab initio simulation program (VASP) using density functional theory (DFT). The non-valent nuclear electrons are

represented by the projected augmented wave method (PAW). All spin polarization calculations were performed by the generalized gradient approximation and the Perdew-Burke-Ernzerhof (PBE) electron exchange-correlation interaction. A kinetic energy cutoff of 480 eV was chosen and the Brillouin zone was sampled in the k-space by a dense grid of 3 × 3 × 5. The atomic positions are relaxed and unconstrained completely until the maximum force on each atom is 0.01 eV $Å^{-1}$. The energy convergence criterion is set to 10^{-5} eV. Grimme's DFT-D2 method is adopted for the calculation of van der Waals interactions. The adsorption energies were estimated via the equation (3):

$$E_{abs} = E_{MOF+H_2/H} - E_{MOF} - E_{H_2/H} \qquad (3)$$

E denotes total energy derived from static DFT calculations. The subscripts $MOF + H_2/H$, MOF and H_2/Hdenote the hydrogen adsorption system, bare perfect framework or defective framework, and free gas molecule or atom, respectively.

Techno-economic Analysis

The objective of this part is to access the techno-economic performance of the materials used for onboard hydrogen storage. A useful life of 1000 cycles and a storage capacity of 5.6 kgH_2 per tank are considered.[2] Neglected are the impacts of internal mass and heat transmission within the storage tanks, as well as heat exchange and insulation between the ambient and the storage system.

The amount of hydrogen stored in the tank is normalized to establish the system-level performance. Consideration includes the tank volume, vessel mass, and storage material mass. The standard energy density of hydrogen is 33.6 kWh/kg. The outcome of the economic study is provided in USD to make an easier comparison for the global market. The price of electronic energy is set at $0.067 per kWh.[3]

The levelized cost of storage (LCOS) in the proposed model is made up of the four cost fractions listed below for representative promising materials: 1. The cost of tank depreciation, C_{tank}; 2. The cost of hydrogen compression, C_{com} ; 3. The cost of heat and/or refrigeration for maintaining temperature, C_{tem}. 4. The cost of hydrogen storage materials depreciation, C_M. Since the price of hydrogen gas is the same for all the storage methods under consideration, it can be disregarded for the sake of comparison in this work. The LCOS is the sum of the four fractions as determined via equation (4):

$$LCOS = C_{tank} + C_{com} + C_{tem} + C_M \qquad (4)$$

For the tank system, it is assumed that the main cost driver is the pressure vessel. C_{tank} is calculated based on the equation (5):

$$C_{tank} = \frac{a_T^P \times x^{b_T^P}}{A_{cycle}} \left(\frac{X_{min}}{X_{min,0}}\right)^{\frac{\log(l_T^P)}{\log 2}} \qquad (5)$$

The fitted parameters of l_T^P, a_T^P and b_T^P are derived from previous work.[4] The A_{cycle}, which reflects the quantity of hydrogen uptake/release cycles, shows how long a tank will last. x represents each tank's storage capacity. A_{cycle} is set to 1000 in this work, and x is set to 5.6 kgH_2.

In the hydrogen compression process, each compression step is followed by a cooling step. It is designed to keep hydrogen stored in various materials at the right temperature. C_{com} is calculated by the multiplication of the electric energy cost c_E (0.06 USD/kWh) and the energy demand for hydrogen processing E_{com}. The C_{com} is determined using equation (6) and (7):

$$C_{com} = c_E \times E_{com} \qquad (6)$$

$$E_{com} = \frac{m}{\eta_{com}}(h_{out} - h_{in}) \qquad (7)$$

h_{out} and h_{in} represent the isentropic enthalpy of output and input hydrogen, respectively. The NIST database is used to retrieve the thermodynamic data. m is the mass flow of hydrogen. The value of η_{com}, which stands for isentropic compression efficiency, is 0.9. The detailed explanation has previously been reported.[4]

For heat and refrigeration, the capital cost and energy cost are determined from the storage temperature. The C_{tem} for TiFeMn, rGO-nanoMg and Pt-de-MgMOF are calculated using equations (8) – (10). While the C_{tem} for MOF-5 is calculated using equations (8), (9) and (11):

$$C_{tem} = c_E \times E_{tem} \qquad (8)$$

$$c_{COP} = \frac{T_0}{T_{RT} - T_0} \qquad (9)$$

$$E_{tem} = \frac{m(h'_{out} - h'_{in})}{c_{COP}} \qquad (10)$$

$$C_{tem} = \frac{e^{2.4647 - 0.01812 \times T_{out}}}{277.778} \qquad (11)$$

c_{COP} is the ideal refrigeration cycle coefficient of performance. T_0 is the lowest thermodynamic temperature of the cold side, T_{RT} is the room temperature. h'_{out} and h'_{in} is the isentropic enthalpy of cooled output hydrogen and input hydrogen, respectively. T_{out} is the desired output temperature. The processes' ability to recover heat is disregarded, and it is assumed that all heat flux is lost to the environment. The detailed description of the parameters and equation can be found in previous studies.[5]

In terms of storage materials, the most explored intermetallic compound, TiFeMn, is selected as the representative material because it has good storage properties.[6] Raw materials cost and gravimetric hydrogen density of TiFeMn have already been reported.[7] The cost of rGO-nanoMg is estimated using an engineering scale-up of synthesis techniques that have been demonstrated in lab.[8] Supplier prices for each of the chemical reagents are gathered. The price for MOF-5 and Mg-MOF-74 is calculated from prior research using actual production methods.[9] Price quotes for chemical reagents are collected from suppliers. C_M is calculated based on the equation (12):

$$C_M = p_m \times \frac{x}{w_m \times A_{cycle}} \qquad (12)$$

p_m is the price of material in USD/kg, w_m is the gravimetric hydrogen density. Each w_m is obtained from previous studies while the w_m for Pt-de-MgMOF is 2.55 wt%.

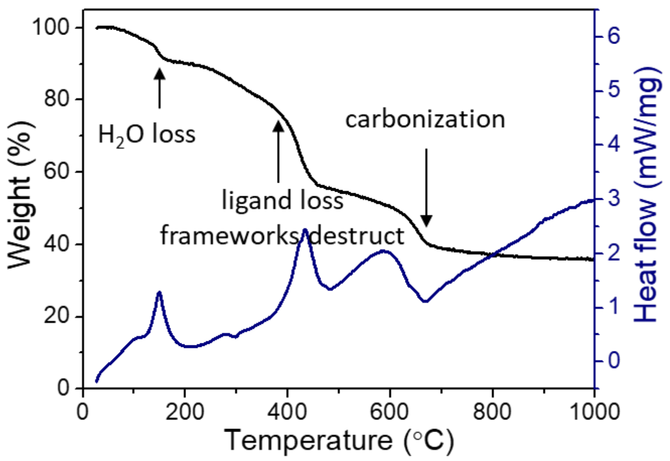

Figure S1. TGA (black line) and DSC (navy line) curves for Mg-MOF-74 under the atmosphere of Ar.

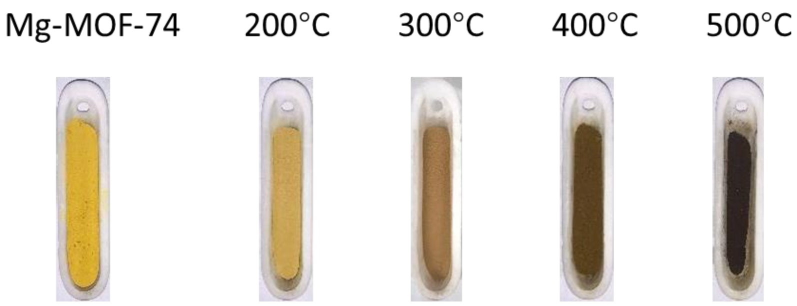

Figure S2. Digital photographs of a series of Mg-MOF-74 after the heat treatment at the indicated temperatures.

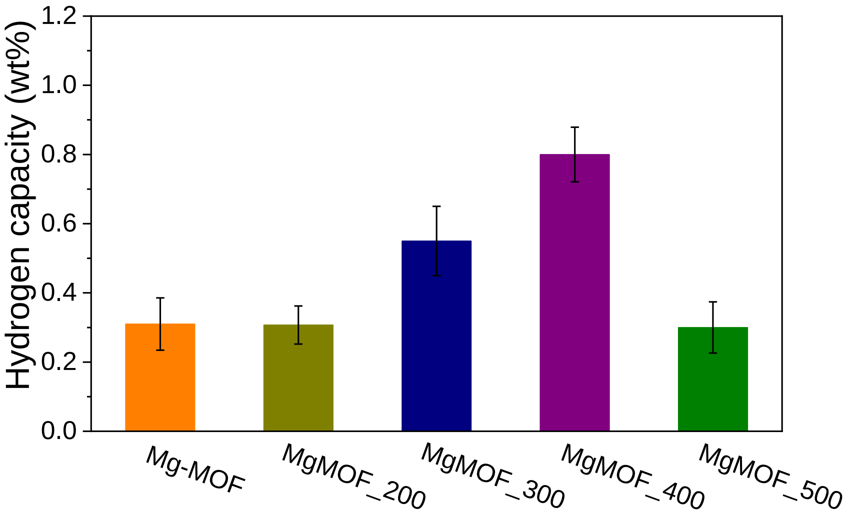

Figure S3. Hydrogen adsorption amount of a series of thermally treated Mg-MOF-74 measured under 50 bar H_2 and 200 °C.

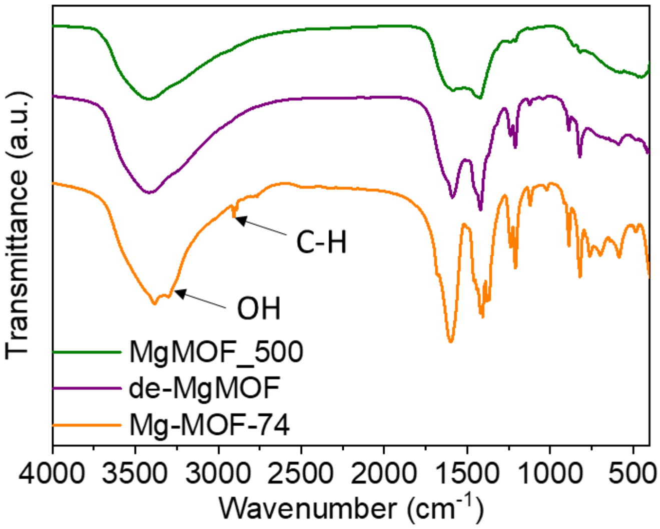

Figure S4. FT-IR results for Mg-MOF-74 and de-MgMOF (Mg-MOF-74 after 400 °C treatment) and MgMOF_500 (Mg-MOF-74 after 500 °C treatment).

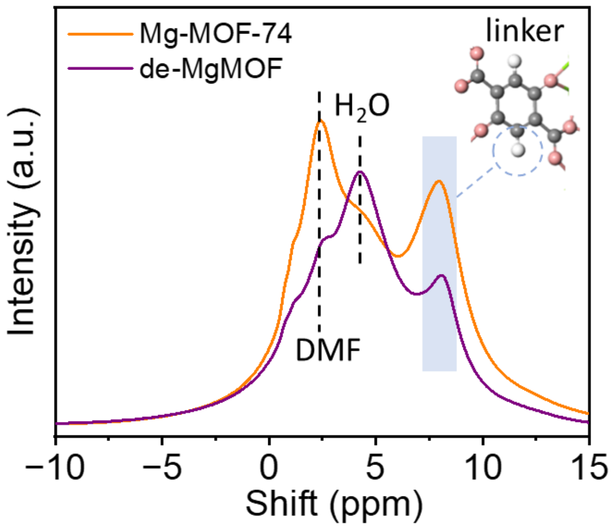

Figure S5. ^{1}H NMR spectra of Mg-MOF-74 and de-MgMOF.

The guest molecules of DMF have been successfully removed after the annealing at 400 °C.[10] In addition, a significant decrease in the resonance peak at 8 ppm (attributed to phenyl hydrogen)[11] indicates partial degradation of the linker.

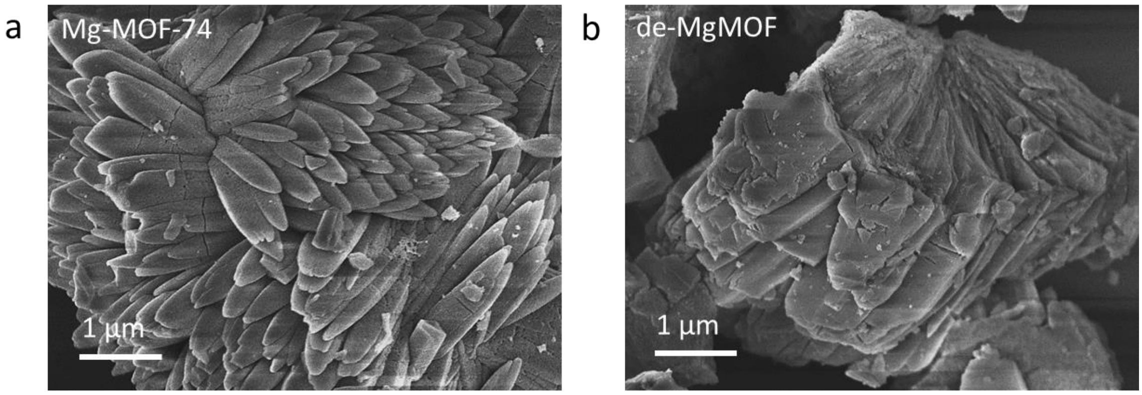

Figure S6. SEM images of (a) Mg-MOF-74 and (b) de-MgMOF.

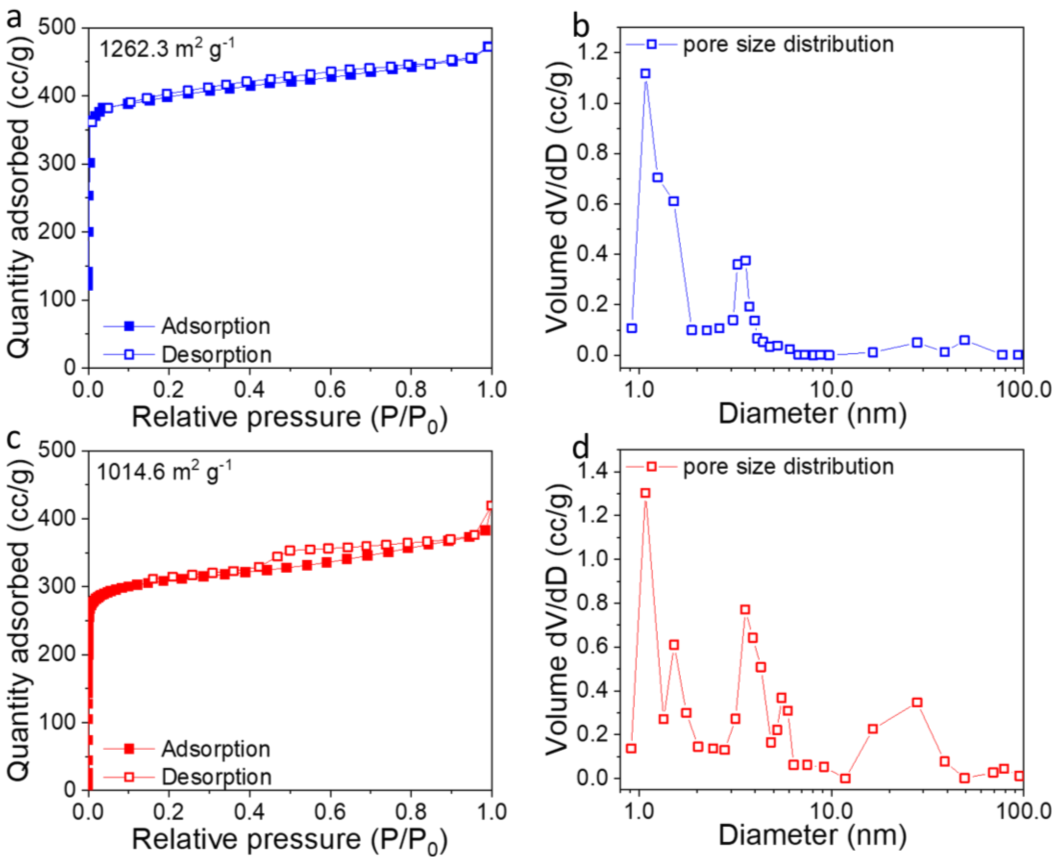

Figure S7. (a) BET isotherm and (b) BJH pore size distributions of Mg-MOF-74. (c) BET isotherm and (d) DFT pore size distributions of de-MgMOF. The open and solid symbols represent the absorption and desorption of the isotherm, respectively.

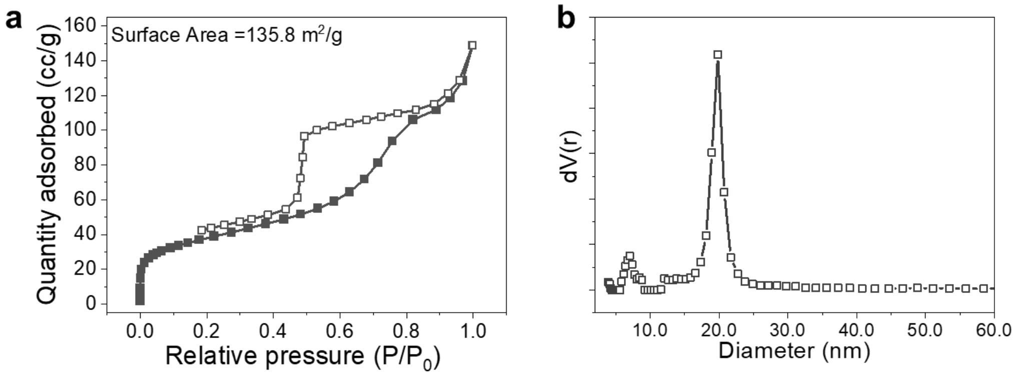

Figure S8. (a) N_2 sorption-desorption isotherms and (b) pore size distribution result of MgMOF_500.

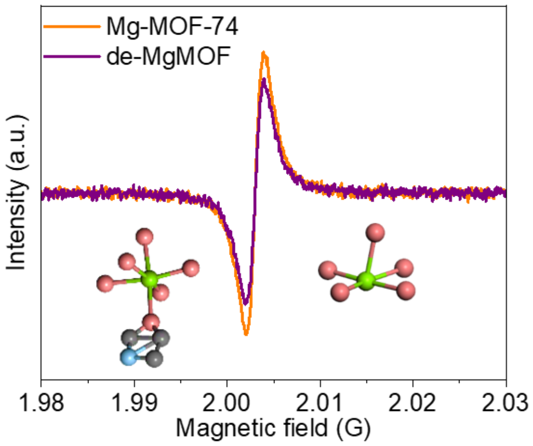

Figure S9. EPR results for Mg-MOF-74 and de-MgMOF obtained at 160 °C.

The EPR signal of de-MgMOF is reduced in intensity by ~20% compared to that of pristine Mg-MOF-74, which indicates the generation of oxygen vacancies and unsaturated Mg sites.

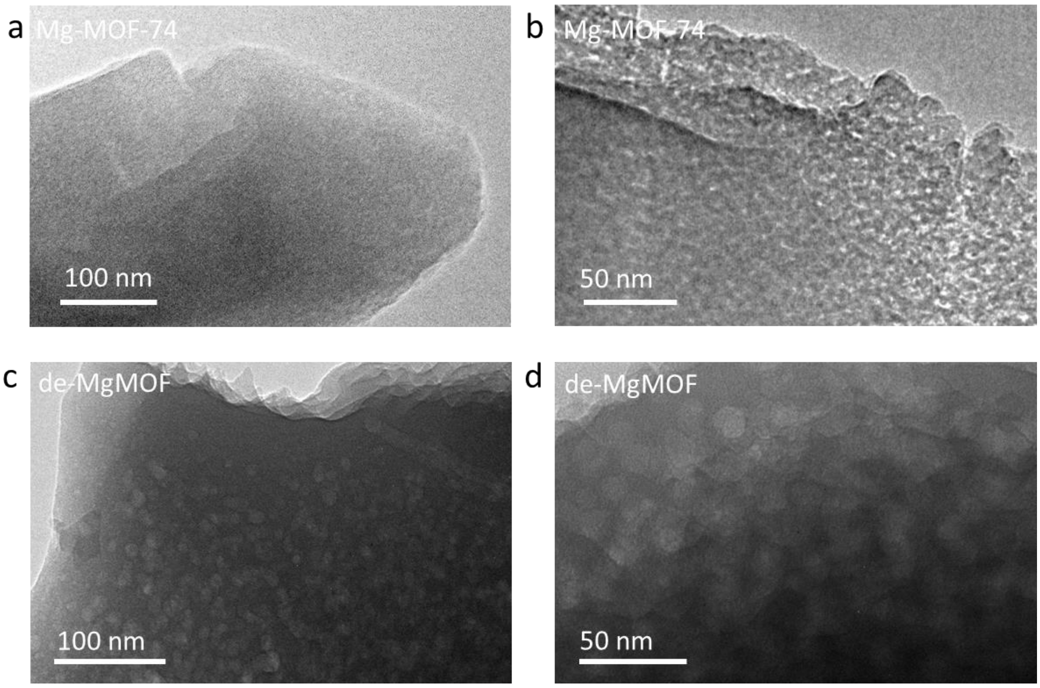

Figure S10. TEM images of (a, b) pristine Mg-MOF-74 and (c, d) de-MgMOF.

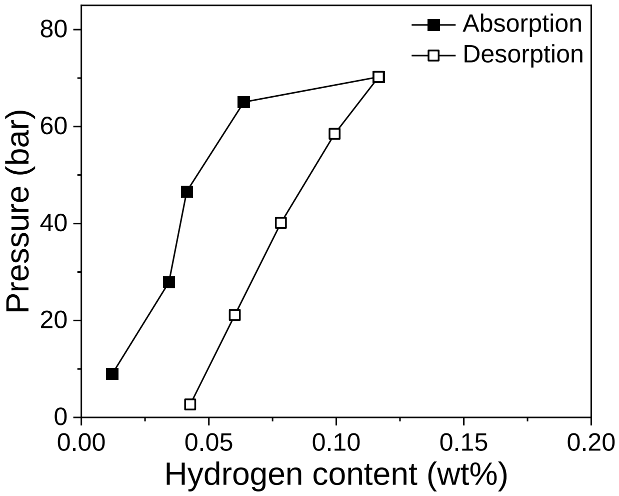

Figure S11. PCT curve of pristine Mg-MOF-74 at 25 °C.

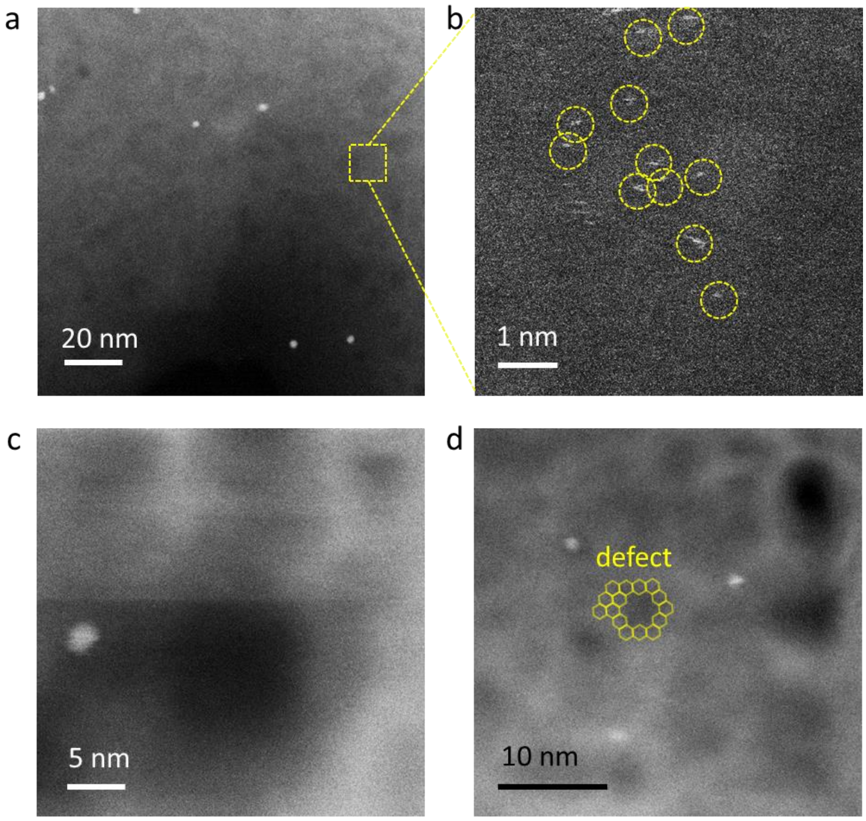

Figure S12. HAADF-STEM images of Pt-de-MgMOF showing (a) Pt clusters and (b) Pt single atoms. (c) and (d) defective framework with linker vacancies.

Although the distribution of Pt clusters in (a) appears to be not uniform, there are a large number of Pt single atoms as shown in (b), proving the existence of the spillover catalyst all over the material.

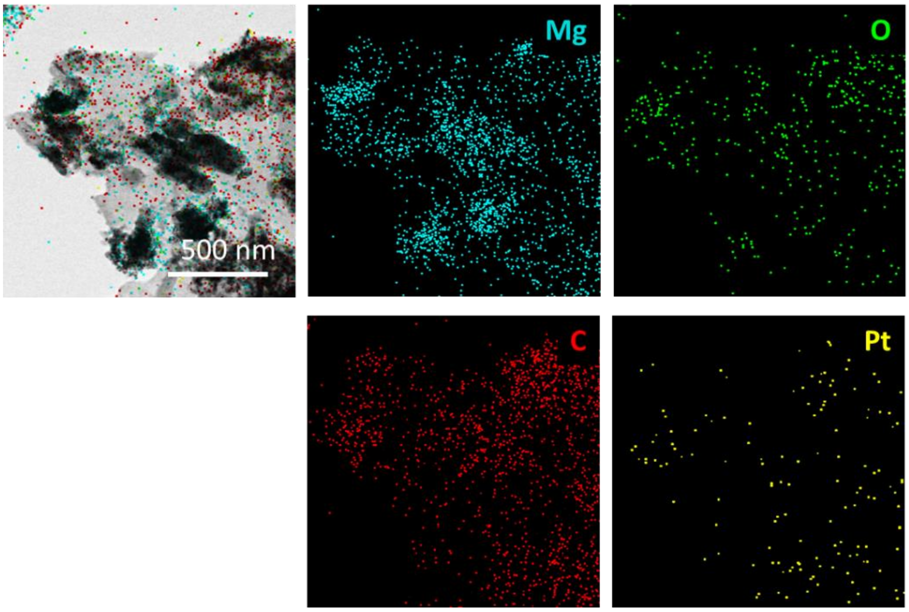

Figure S13. EDS mapping images of Pt-de-MgMOF, showing the uniform distribution of Pt.

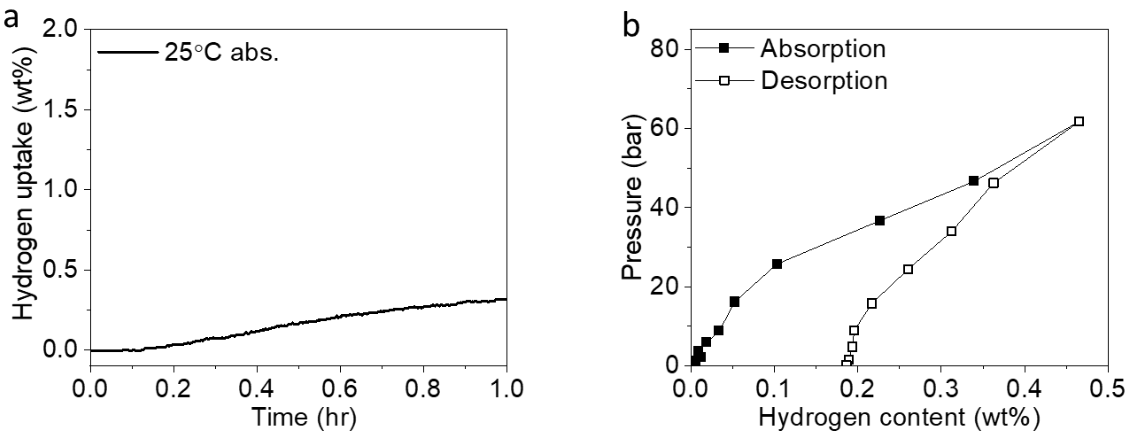

Figure S14. (a) Hydrogen absorption kinetics and (b) PCT curve of Pt-de-MgMOF at 25°C.

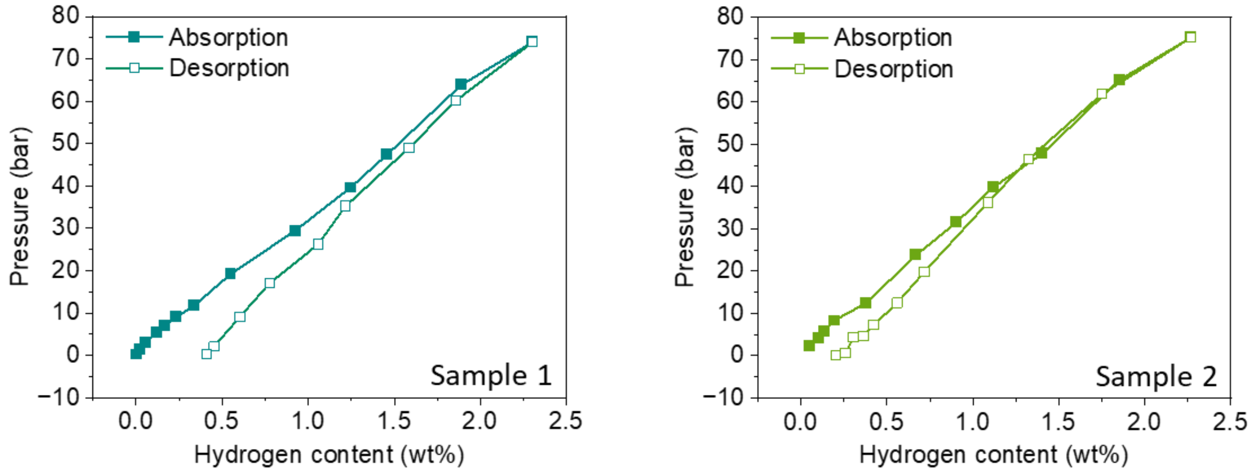

Figure S15. PCT curves of the other two batches of Pt-de-MgMOF at 160 °C.

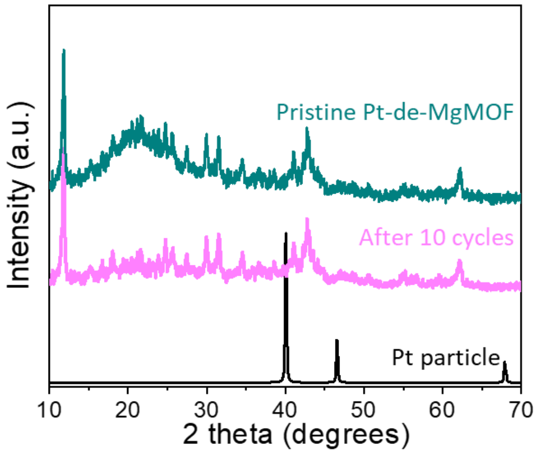

Figure S16. XRD patterns of Pt-de-MgMOF before and after 10 cycles of hydrogen adsorption/desorption at 160 °C, with a reference of Pt nanoparticles.

The diffraction peaks of Pt nanoparticles are not observable on Pt-de-MgMOF.

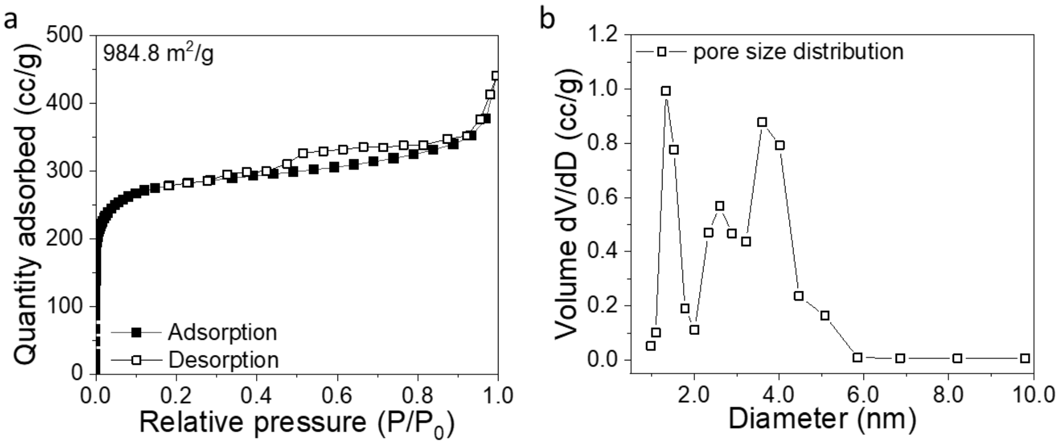

Figure S17. (a) BET isotherm measured at 77 K using liquid N_2, and (b) the pore size distribution of Pt-de-MgMOF after 10 cycles of hydrogen adsorption/desorption.

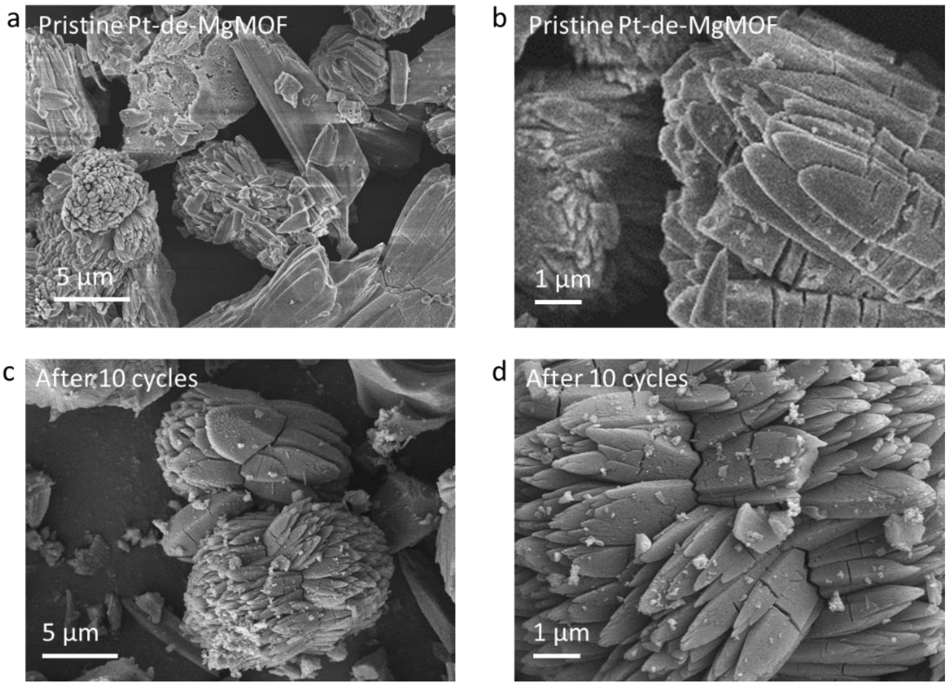

Figure S18. SEM images of (a, b) pristine Pt-de-MgMOF and (c, d) Pt-de-MgMOF after 10 cycles of hydrogen adsorption/desorption.

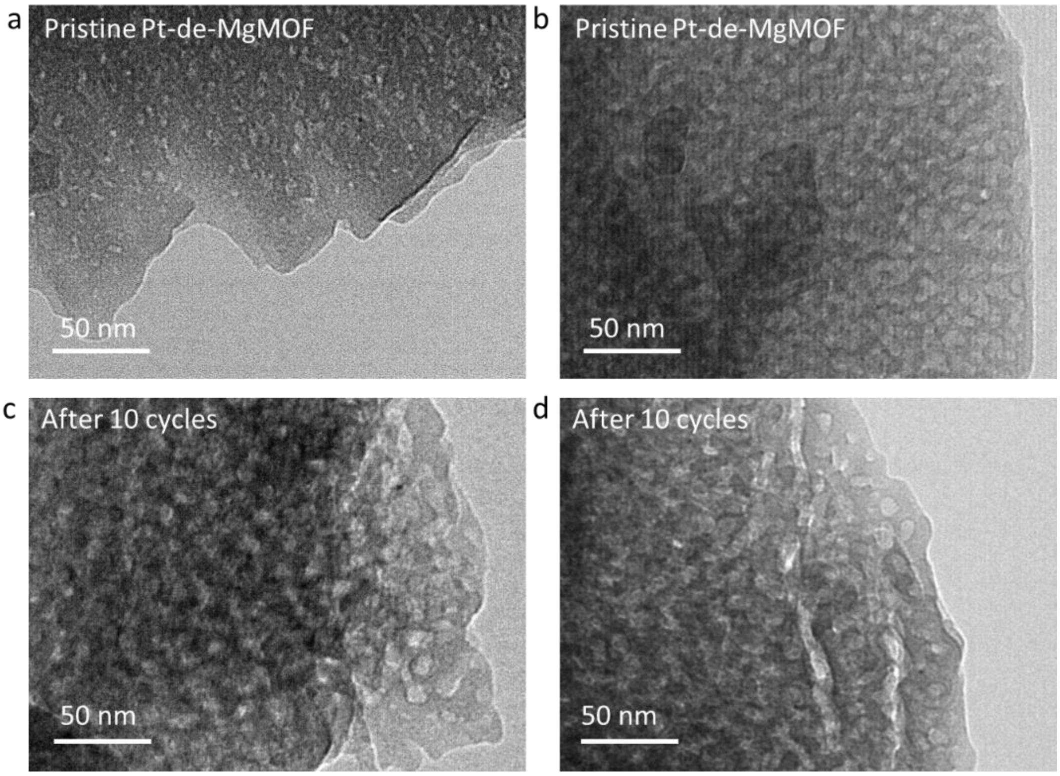

Figure S19. TEM images of (a, b) pristine Pt-de-MgMOF and (c, d) Pt-de-MgMOF after 10 cycles of hydrogen adsorption/desorption.

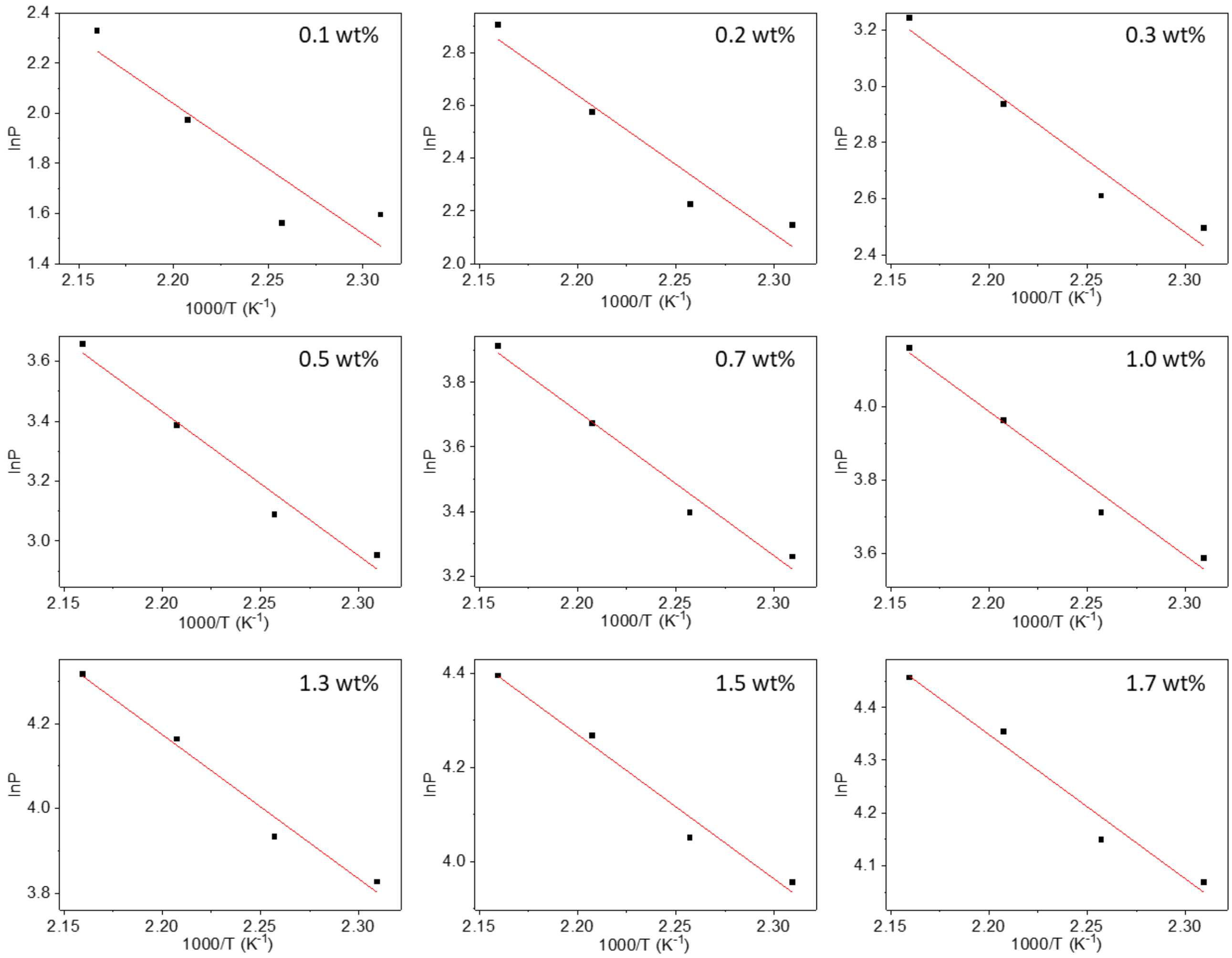

Figure S20. lnP versus 1000/T plots of Pt-de-MgMOF under different hydrogen contents.

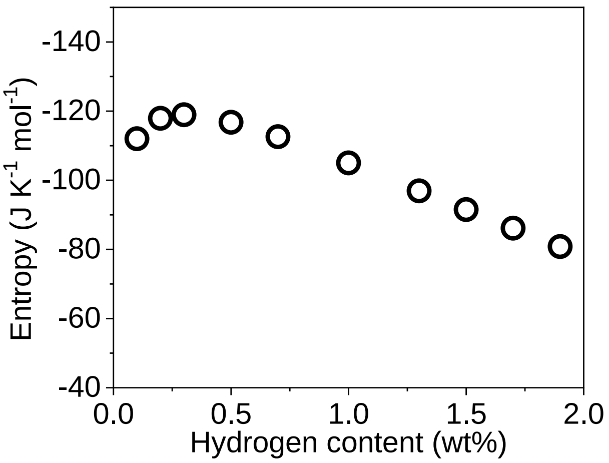

Figure S21. Calculated entropy change of Pt-de-MgMOF as a function of the hydrogen content.

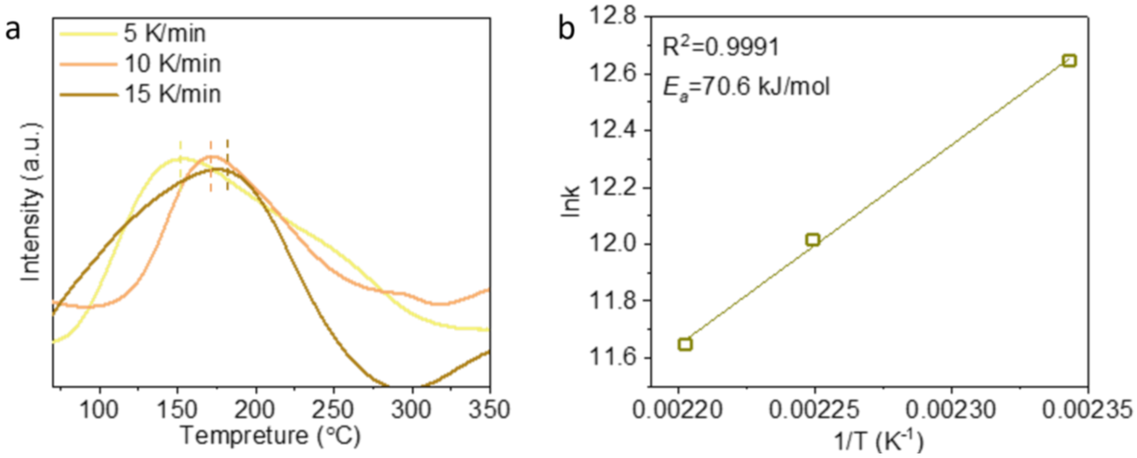

Figure S22. (a) TPD results of hydrogenated Pt-de-MgMOF under different heating rates. (b) Arrhenius plot with the calculated activation energy (E_a).

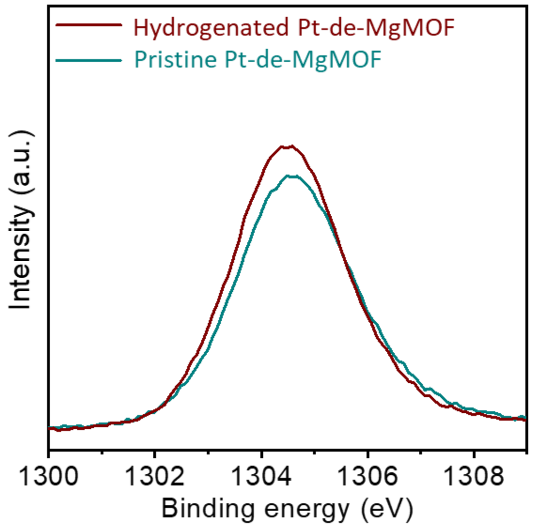

Figure S23. Mg 1s XPS spectra of the pristine and hydrogenated Pt-de-MgMOF.

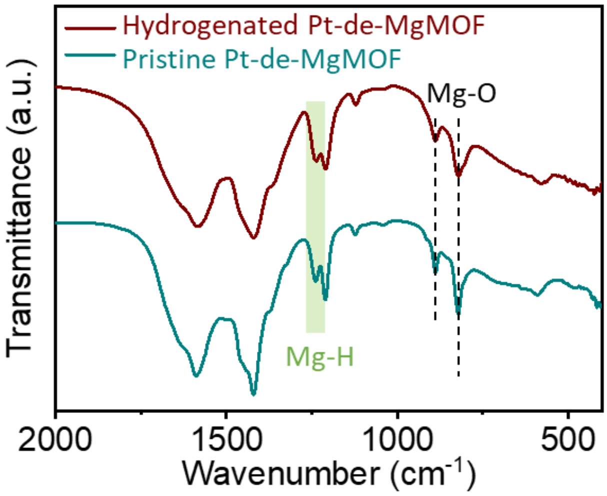

Figure S24. FT-IR results for pristine and hydrogenated Pt-de-MgMOF.

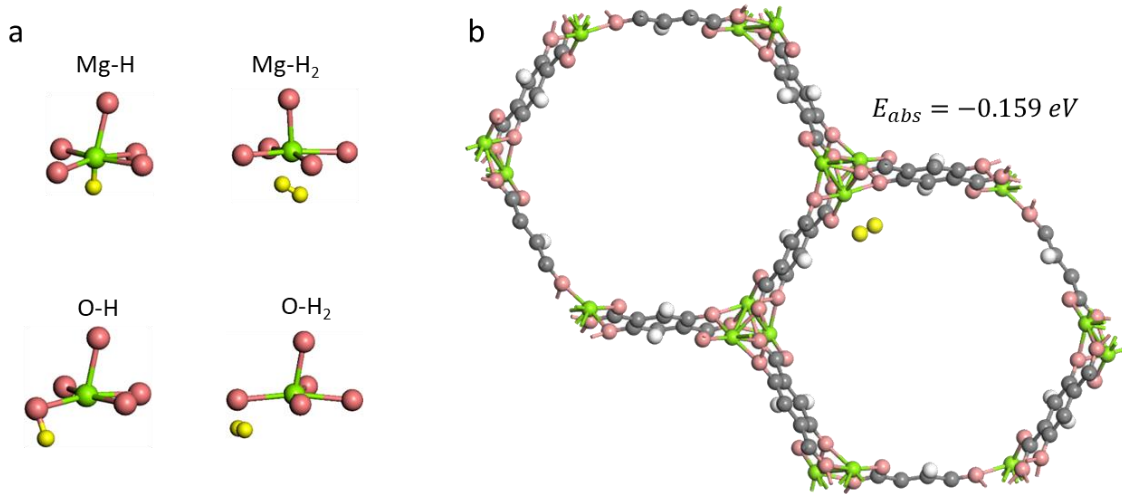

Figure S25. (a) Schematic illustration of the hydrogen adsorption on an MgO_5 knot. (b) Calculated configuration and hydrogen adsorption energy (E_{ads}) when a molecular hydrogen is absorbed on the desolvated Mg-MOF-74.

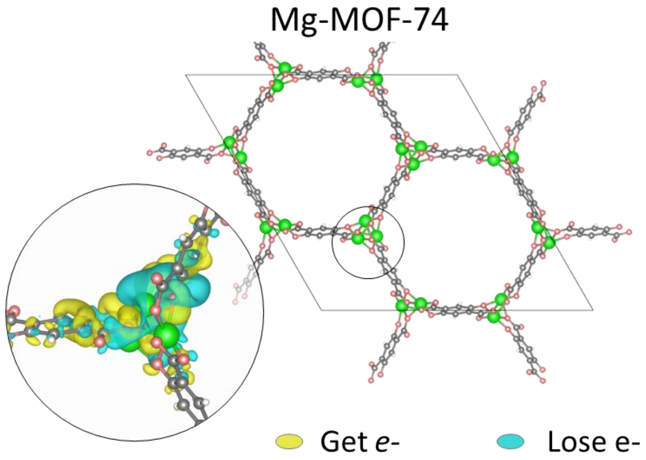

Figure S26. The charge density differences of Mg-MOF-74 and de-MgMOF are plotted with an isovalue of 0.2×10^{-3} e $Å^{-1}$. The charge accumulation and depletion are colored in yellow and cyan, respectively.

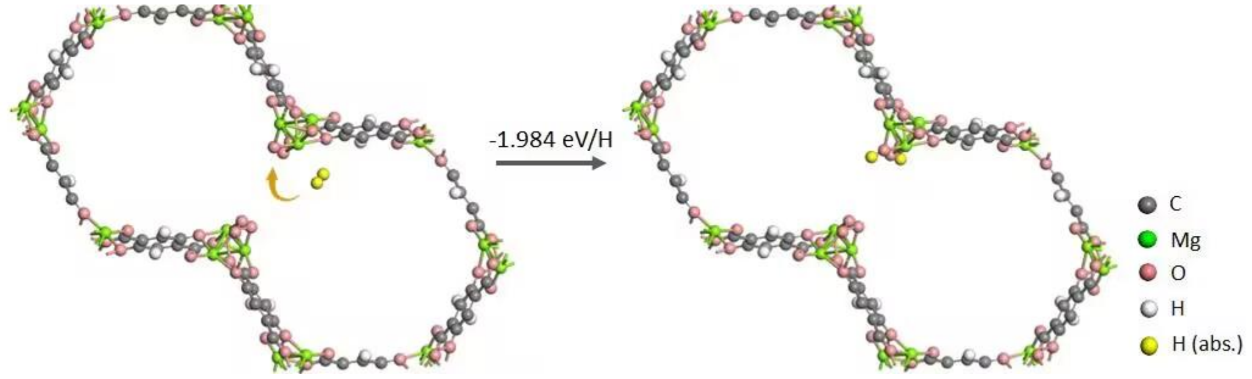

Figure S27. Activation of a H_2 molecule on the Mg_3O_9 cluster of de-MgMOF.

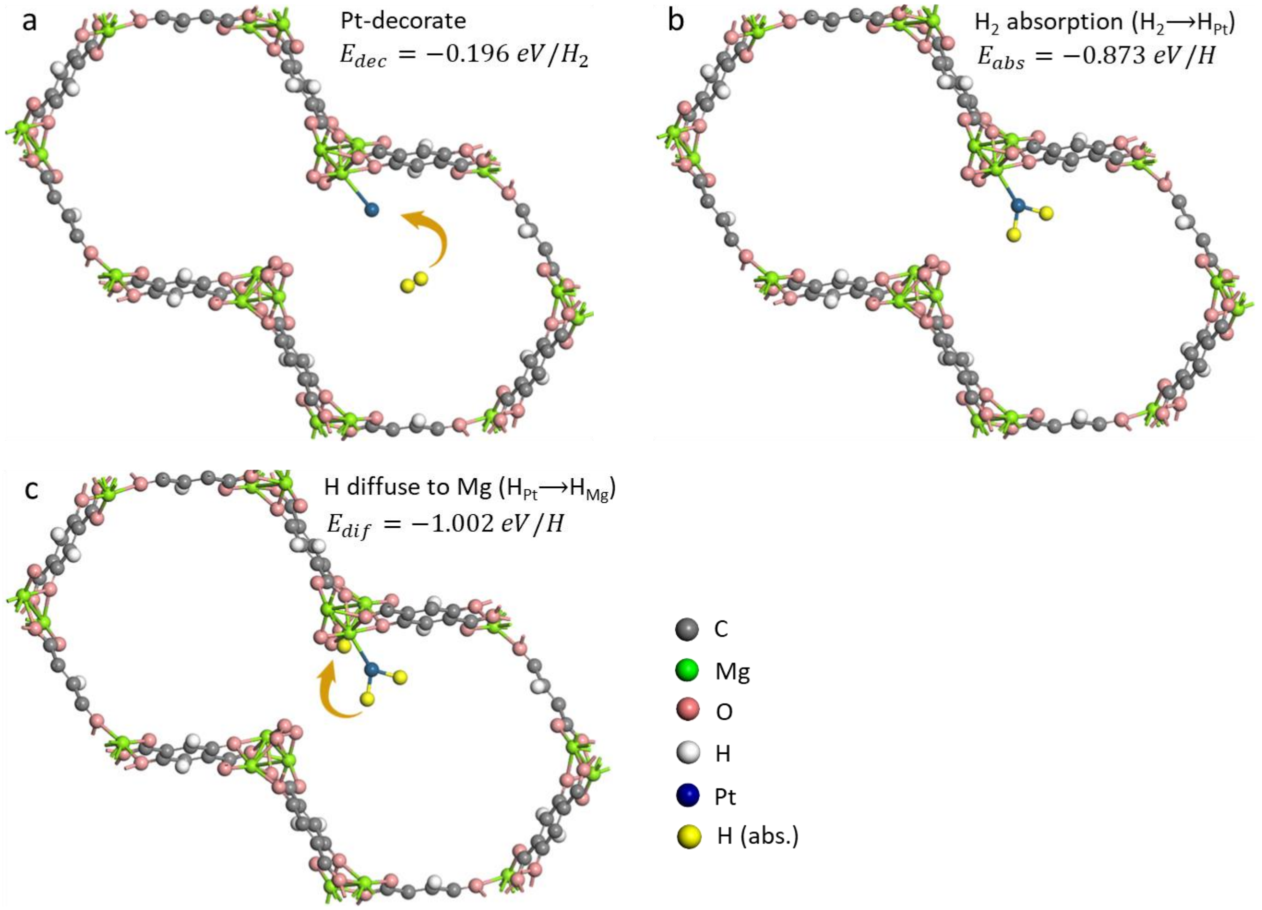

Figure S28. Calculation results for hydrogen spillover on Pt-de-MgMOF. (a) Pt-de-MgMOF with an H_2 molecular. (b) H_2 molecule dissociated into two H atoms on Pt catalyst. (c) one H atom moves to the Mg atom close to the Pt.

H_{Pt} refers to the hydrogen bonded to Pt, and H_{Mg} refers to the hydrogen bonded to Mg. Firstly, the H_2 molecule is adsorbed and dissociates spontaneously on the Pt catalyst to form H_{Pt}. Then, the H atom diffuses to Mg atom to form H_{Mg} and another H_2 molecule will absorb on Pt. It can be concluded that the spillover process of hydrogen absorption is exothermic.

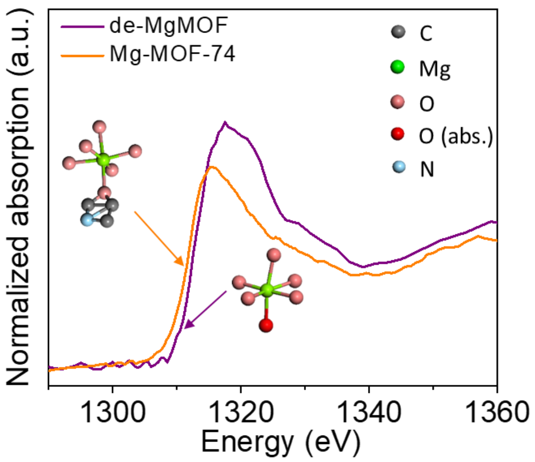

Figure S29. XANES spectra of Mg-MOF-74 and de-MgMOF.

The samples are exposed to air while conducting the test.

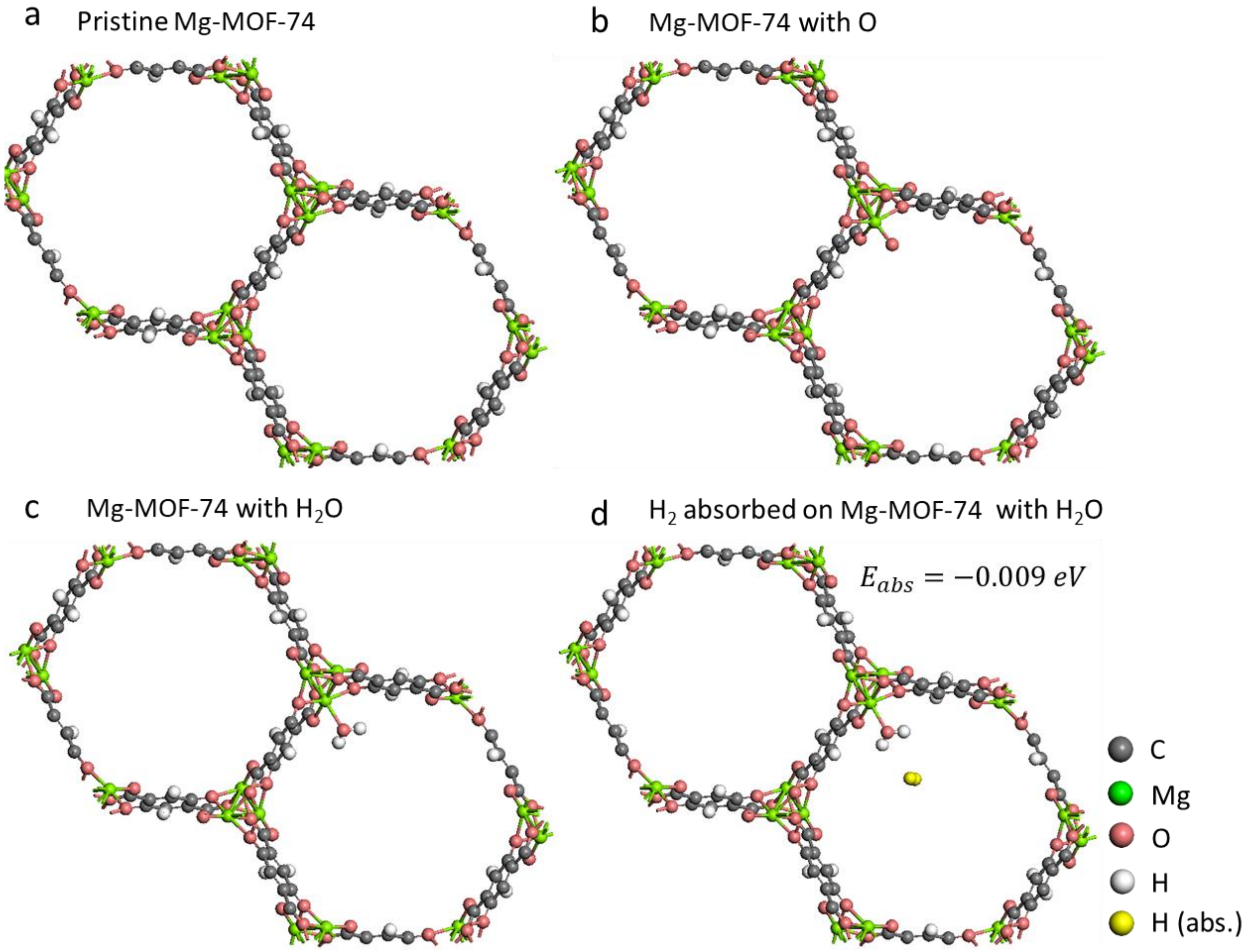

Figure S30. Calculated configurations of (a) perfect desolvated Mg-MOF-74, (b) oxygen and (c) H_2O absorbed on desolvated Mg-MOF-74. (d) H_2 molecule absorbed on desolvated Mg-MOF-74 with a H_2O attached.

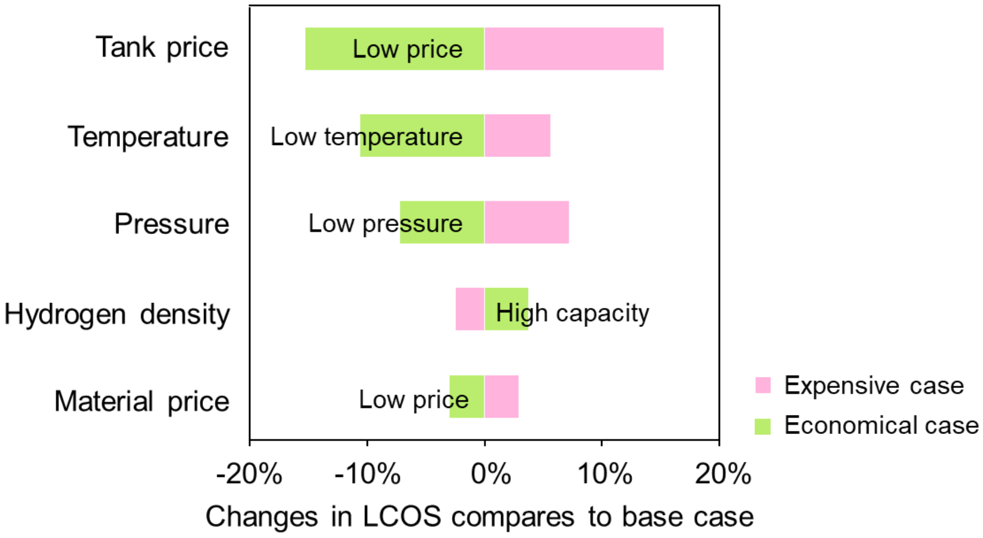

Figure S31. Tornado chart showing parameter sensitivity of Pt-de-MgMOF in the system level.

Table S1. ICP-OES results for pristine Pt-de-MgMOF, hydrogenated Pt-de-MgMOF (at 60 bar, 160 °C), and Pt-de-MgMOF after being stored in ambient air for 2 weeks.

Sample	Mg (wt%)	Pt (wt%)
Pristine Pt-de-MgMOF	16.87	0.36
Hydrogenated Pt-de-MgMOF	16.91	0.35
Pt-de-MgMOF after stored for 2 weeks	16.63	0.33

Table S2. Structural parameters for the Pt L-edge EXAFS fitting of pristine (S_0^2=0.90) and hydrogenated (S_0^2=0.98) Pt-de-MgMOF.

Sample	Scattering pair	CN	*R* (Å)	σ^2 (Å)	ΔE_0 (eV)	R factor
Pristine Pt-de-MgMOF	Pt-Pt	10.77	2.77	0.007	8.364	0.06
	Pt-Mg	0.35	3.27	0.010		
Hydrogenated Pt-de-MgMOF	Pt-Pt	9.65	2.76	0.006	8.349	0.03
	Pt-Mg	0.99	3.16	0.009		

Table S3. Hydrogen storage capacity of MOFs with chemisorption potential.

Absorbent	Catalyst	Temperature (K)	Pressure (bar)	Capacity (wt%)	Ref.
Fe-BTT	/	298	96	1.05	[12]
UIO-66	/	298	100	0.81	[13]
Cr-MIL-101	Li18Crown6	298	170	0.65	[14]
MIL-100(Al)	Pd	298	86	0.60	[15]
IRMOF-8	/	298	90	0.41	[16]
IRMOF-8	Pt	298	96	0.82	[16]
MOF-5	/	298	65	0.28	[17]
$Cu_3(BTC)_2$	/	298	65	0.35	[17]
Co_2(*m*-dobdc)	/	298	100	0.95	[18]
Mn-MOF-1m’	/	298	90	1.46	[19]
HKUST-1	/	303	35	0.47	[20]
		353	35	0.35	
$Zn_4O(BDC)_3$	/	298	83	1.25	[21]
de-MgMOF	Pt	433	81	2.55	This work

Table S4. Fitting parameters to the exponential equation for H_2 adsorption of Pt-de-MgMOF at 160 °C, 170 °C, 180 °C, and 190 °C.

Model	Exponential			
Equation	$y = y_0 + A*\exp(R_0*x)$			
Plot	160°C ads.	170°C ads.	180°C ads.	190°C ads.
y_0	358.61981 ± 130.31888	168.82114 ± 9.67471	150.83413 ± 8.8434	116.61936 ± 10.45352
A	-357.34308 ± 130.04146	-168.65352 ± 9.52968	-149.83668 ± 8.61612	-115.00303 ± 10.02746
R_0	-0.10264 ± 0.04101	-0.2764 ± 0.02018	-0.42212 ± 0.03507	-0.78078 ± 0.11128
Reduced Chi-Sqr	0.7127	0.19943	0.73092	2.52031
R-Square (COD)	0.99856	0.99966	0.99918	0.99772
Adj. R-Square	0.99833	0.99961	0.99906	0.9964

Table S5. TPD results of Pt-de-MgMOF that hydrogenated at 1 bar and 160 °C.

Peak Number	Temperature at Maximum (°C)	Quantity (mmol g^{-1})	Hydrogen desorbed (wt%)
1	147.9	2.255592	0.45
2	231.5	0.864889	0.17

References

[1] R. L. Blaine, H. E. Kissinger, *Thermochim. Acta* **2012**, 540, 1.

[2] a)B. D. James, C. Houchins, J. M. Huya-Kouadio, D. A. DeSantis, Department of Energy, 2016; b)M. D. Paster, R. K. Ahluwalia, G. Berry, A. Elgowainy, S. Lasher, K. McKenney, M. Gardiner, *Int. J. Hydrogen Energy* **2011**, 36, 14534.

[3] U.S. Energy Information Administration, U.S. Energy Information Administration 2021.

[4] J. Jepsen, J. M. Bellosta von Colbe, T. Klassen, M. Dornheim, *Int. J. Hydrogen Energy* **2012**, 37, 4204.

[5] a)W. L. Luyben, *Computers & Chemical Engineering* **2017**, 103, 144; b)K. A. G. Amankwah, J. S. Noh, J. A. Schwarz, *Int. J. Hydrogen Energy* **1989**, 14, 437; c)P. Peng, A. Anastasopoulou, K. Brooks, H. Furukawa, M. E. Bowden, J. R. Long, T. Autrey, H. Breunig, *Nat. Energy* **2022**, 7, 448.

[6] I. A. Hassan, H. S. Ramadan, M. A. Saleh, D. Hissel, *Renewable Sustainable Energy Rev.* **2021**, 149, 111311.

[7] P. Feng, Y. Liu, I. Ayub, Z. Wu, F. Yang, Z. Zhang, *Appl. Energy* **2019**, 242, 148.

[8] E. S. Cho, A. M. Ruminski, Y.-S. Liu, P. T. Shea, S. Kang, E. W. Zaia, J. Y. Park, Y.-D. Chuang, J. M. Yuk, X. Zhou, T. W. Heo, J. Guo, B. C. Wood, J. J. Urban, *Adv. Funct. Mater.* **2017**, 27, 1704316.

[9] D. DeSantis, J. A. Mason, B. D. James, C. Houchins, J. R. Long, M. Veenstra, *Energy & Fuels* **2017**, 31, 2024.

[10] a)L. Feng, K. Y. Wang, G. S. Day, M. R. Ryder, H. C. Zhou, *Chem. Rev.* **2020**, 120, 13087; b)X. Liang, P. Wang, C. Li, M. Yuan, Q. Shi, J. Dong, *Microporous Mesoporous Mater.* **2021**, 320, 111109.

[11] S. Chen, S. Mukherjee, B. E. G. Lucier, Y. Guo, Y. T. A. Wong, V. V. Terskikh, M. J. Zaworotko, Y. Huang, *J. Am. Chem. Soc.* **2019**, 141, 14257.

[12] K. Sumida, S. Horike, S. S. Kaye, Z. R. Herm, W. L. Queen, C. M. Brown, F. Grandjean, G. J. Long, A. Dailly, J. R. Long, *Chem. Sci.* **2010**, 1, 184.

[13] S. E. Bambalaza, H. W. Langmi, R. Mokaya, N. M. Musyoka, L. E. Khotseng, *ACS Appl. Mater. Interfaces* **2020**, 12, 24883−24894.

[14] G. Orcajo, H. Montes-Andrés, J. A. Villajos, C. Martos, J. A. Botas, G. Calleja, *Int. J. Hydrogen Energy* **2019**, 44, 19285.

[15] C. Zlotea, R. Campesi, F. Cuevas, E. Leroy, P. Dibandjo, C. Volkringer, T. Loiseau, G. Férey, M. Latroche, *J. Am. Chem. Soc.* **2010**, 132, 2991−2997.

[16] L. Wang, N. R. Stuckert, H. Chen, R. T. Yang, *J. Phys. Chem. C* **2011**, 115, 4793−4799.
[17] B. Panella, M. Hirscher, H. Pütter, U. Müller, *Adv. Funct. Mater.* **2006**, 16, 520.
[18] M. T. Kapelewski, T. Runcevski, J. D. Tarver, H. Z. H. Jiang, K. E. Hurst, P. A. Parilla, A. Ayala, T. Gennett, S. A. FitzGerald, C. M. Brown, J. R. Long, *Chem. Mater.* **2018**, 30, 8179.
[19] M. Dincǎ, A. Dailly, Y. Liu, C. M. Brown, D. A. Neumann, J. R. Long, *J. Am. Chem. Soc.* **2006**, 128, 16876.
[20] K.-S. Lin, A. K. Adhikari, C.-N. Ku, C.-L. Chiang, H. Kuo, *Int. J. Hydrogen Energy* **2012**, 37, 13865−13871.
[21] S. S. Kaye, A. Dailly, O. M. Yaghi, J. R. Long, *J. Am. Chem. Soc.* **2007**, 129, 14176−14177.
